# Microarray analysis of RNA extracted from formalin-fixed, paraffin-embedded and matched fresh-frozen ovarian adenocarcinomas

**DOI:** 10.1186/1755-8794-2-23

**Published:** 2009-05-08

**Authors:** Grazyna Fedorowicz, Steve Guerrero, Thomas D Wu, Zora Modrusan

**Affiliations:** 1 Department of Molecular Biology, Genentech, Inc., 1 DNA Way, South San Francisco, CA 94080, USA; 2 Department of Bioinformatics, Genentech, Inc., 1 DNA Way, South San Francisco, CA 94080, USA

## Abstract

**Background:**

Gene expression profiling of formalin-fixed, paraffin-embedded (FFPE) samples represents a valuable approach for advancing oncology diagnostics and enhancing retrospective clinical studies; however, at present, this methodology still requires optimization and thus has not been extensively used. Here, we utilized thorough quality control methods to assess RNA extracted from FFPE samples and then compared it to RNA extracted from matched fresh-frozen (FF) counterparts. We preformed genome-wide expression profiling of FF and FFPE ovarian serous adenocarcinoma sample pairs and compared their gene signatures to normal ovary samples.

**Methods:**

RNA from FFPE samples was extracted using two different methods, Ambion and Agencourt, and its quality was determined by profiling starting total RNA on Bioanalyzer and by amplifying increasing size fragments of *beta actin *(*ACTB*) and *claudin 3 *(*CLDN3*) by reverse-transcriptase polymerase chain reaction. Five matched FF and FFPE ovarian serous adenocarcinoma samples, as well as a set of normal ovary samples, were profiled using whole genome Agilent microarrays. Reproducibility of the FF and FFPE replicates was measured using Pearson correlation, whereas comparison between the FF and FFPE samples was done using a Z-score analysis.

**Results:**

Data analysis showed high reproducibility of expression within each FF and FFPE method, whereas matched FF and FFPE pairs demonstrated lower similarity, emphasizing an inherent difference between the two sample types. Z-score analysis of matched FF and FFPE samples revealed good concordance of top 100 differentially expressed genes with the highest correlation of 0.84. Genes characteristic of ovarian serous adenocarcinoma, including a well known marker *CLDN3*, as well as potentially some novel markers, were identified by comparing gene expression profiles of ovarian adenocarcinoma to those of normal ovary.

**Conclusion:**

Conclusively, we showed that systematic assessment of FFPE samples at the RNA level is essential for obtaining good quality gene expression microarray data. We also demonstrated that profiling of not only FF but also of FFPE samples can be successfully used to identify differentially expressed genes characteristic of ovarian carcinoma.

## Background

According to the American Cancer Society, ovarian cancer is the fifth leading cause of cancer deaths in women in the United States. The most common, epithelial, type of ovarian cancer can be divided into several subtypes including: serous, endometrioid, mucinous, clear cell and undifferentiated. Serous adenocarcinoma comprises majority of cases and exhibits a poor 5-year survival rate. Up to 90% of ovarian cancers might be cured if identified in an early stage. When diagnosed in later stages, the rate drops significantly to a range of 30–50%. Detection of ovarian cancer is often delayed or missed because of a lack of clear symptoms and absence of reliable diagnostic methods. Cancer marker 125 (CA125), the product of *mucin 16*, is currently used for testing patients with elevated risk of ovarian cancer. However, this marker alone does not provide the required sensitivity or specificity to detect all cases [1]. Another gene, *claudin 3 *(*CLDN3*), has been found to be highly expressed at gene and protein levels and thus has been suggested as a reliable marker of ovarian cancer [2-5].

Large repositories of formalin-fixed, paraffin-embedded (FFPE) samples are available and could be used to identify markers for diagnosis of many diseases. While tissue integrity in FFPE specimens is often better preserved than in matched fresh-frozen (FF) counterparts, the quality of nucleic acids in FFPE samples is far from optimal due to chemical crosslinking and nucleic acid fragmentation [6-8]. Despite the detrimental effect of the fixative, numerous studies using archived FFPE samples have generated satisfactory reverse-transcriptase polymerase chain reaction (RT-PCR) data [9-13]. Recently, a number of genome-wide microarray studies has been conducted to investigate gene expression in FFPE samples or to compare the performance of FFPE samples with their matched FF counterparts [14-23]. While the results of some studies are discouraging [16,19], many archived FFPE samples have been successfully used to identify prognostic and diagnostic gene signatures for numerous diseases, including various carcinomas [21-24].

## Methods

### Samples

Matched FF and FFPE samples were obtained from five ovarian serous adenocarcinoma patients. Samples 3136, 3138, 3194 and 3207 were collected on 11/2004, 11/2004, 05/2005, and 06/2005, respectively. A portion of each sample was either frozen at -80°C until extraction or fixed within 30 minutes of surgery by incubation in 10% neutral-buffered formalin (NBF) for 4–18 hours at 4°C. Patient sample 390 was collected on 01/2005 and was either frozen or fixed for 24 hours at room temperature in 10% NBF within 30 minutes of surgery. Only tumor samples containing minimal necrosis (<10%) and consisting of 70% or more tumor cells were used in this study. A set of normal ovary samples was obtained from different patients by dissecting normal tissue adjacent to tumors. All tumor and normal ovary samples were acquired commercially by the Human Tissue Lab at Genentech.

### RNA extraction methods

#### FF samples

Three 10-micron sections were homogenized individually and RNA was extracted using the RNeasy Lipid Tissue Mini Kit (Qiagen, San Diego, CA). Replicate RNA preps were pooled to obtain sufficient amounts of starting material. For normal ovary samples, RNA was extracted using Qiagen's ALLPrep method that included on-column DNase treatment.

#### FFPE samples

After evaluating four FFPE RNA extraction methods (Invitrogen PureLink, Ambion RecoverAll, Ambion Optimum and Agencourt FormaPure; data not shown), the Optimum FFPE RNA Isolation Kit (Ambion, Austin, TX) and the FormaPure Kit (Agencourt, Beverly, MA) were selected for this study based on their potential to generate the most abundant population of high molecular weight RNA fragments. The two methods were used to extract RNA from five archived FFPE ovarian serous adenocarcinoma samples. Up to eight 10-micron FFPE sections were processed per patient. Ambion's RNA extraction procedure was optimized for maximum RNA recovery (Susanna Stinson, Genentech, Inc., personal communication) by elevating the temperature of the first 10 min deparaffinization to 55°C and digesting the samples for 3 hours at 55°C. After adding a fresh aliquot of Proteinase K, the samples were digested for an additional hour at 55°C. The Agencourt protocol was followed without any modifications. DNase treatment was applied to both FF and FFPE samples and was followed by phenol:chloroform:isoamyl alcohol purification and ethanol precipitation.

### Quality control methods

The quantity of RNA and labeled cRNA was measured using Nanodrop ND-1000 UV-spectrophotometer (NanoDrop Technologies, Wilmington, DE). Specific activity of cRNA, calculated as picomoles of Cy5 dye per microgram (μg) of cRNA, was also measured by Nanodrop. Sample integrity was evaluated by profiling both RNA and cRNA on Agilent 2100 Bioanalyzer (Agilent Technologies, Santa Clara, CA).

RNA integrity was determined by amplifying different length fragments (~200, 400, 600 and 800 basepairs (bp)) of *beta actin *(*ACTB*) and *CLDN3 *genes using Qiagen's OneStep RT-PCR kit protocol. The following 5'-3' primers were used to amplify *ACTB *[GenBank:NM_001101]: 200 F GGTGATAGCATTGCTTTCGTGTAA, 400 F CAGTCGGTTGGAGCGAGCATCC, 600 F CTCCATCGTCCACCGCAAATGC, 800 F GGCACCACCATGTACCCTGGCA and R TCAAGTCAGTGTACAGGTAAGCC. The following 5'-3' primers were used to amplify *CLDN3 *[GenBank:NM_001306]: 200 F CCATCCAGCGTGCAGCCTTGC, 400 F GCTGCTCTGCTGCTCGTGTCC, 600 F CCAAGATCACCATCGTGGCAGG, 800 F GCCTGTGGATGAACTGCGTGG and R AGTATTGGCGGTCACCCAGGC. Five nanograms of RNA were used as a template for reverse transcription (30 min at 50°C), followed by activation of the HotStarTaq polymerase (15 min at 95°C), 35 cycles of PCR (30 sec at 95°C, 30 sec at 60°C, and 1 min at 72°C), and final 10 min extension at 72°C. Each fragment was amplified individually; however, all of them were pooled and electrophoresed in a single lane. PCR products were visualized on 4% agarose e-gels (Invitrogen, Carlsbad, CA). SimplyLoad 100 bp DNA Ladder (Lonza, Basel, Switzerland) was used to determine product size.

### Microarrays

Total RNA was labeled according to Agilent's Low RNA Input Fluorescent Linear Amplification Kit. The test samples (i.e. RNA from FF and FFPE samples) were labeled with the Cy5 dye and the reference sample (i.e. Universal Human Reference, Stratagene, La Jolla, CA) was labeled with the Cy3 dye. Matched FF samples (5 patients, 2 replicates) and FFPE samples extracted using either the Agencourt method (5 patients, 3 replicates for 3136, 3138 and 3194, 2 replicates for 3207 and 390) or the Ambion method (5 patients, no replicates) were hybridized to Agilent Whole Human Genome 4×44K microarrays according to the manufacturer's protocol. Microarray images were analyzed using Agilent's Feature Extraction (FE) software, version 9.5.

### Data analysis

Reproducibility of the FF and FFPE sample types was measured using Pearson correlations (r). Only "passing" probes were included in this analysis, where "passing" consisted of probes that were not recognized as outliers by the FE software, were significantly above background, and had a P value of the Cy5/Cy3 ratio below 0.05. We compared results between sample types (frozen versus fixed) by applying a Z-score analysis [25] to the four samples whose quality was adequate for microarray profiling (3136, 3138, 3194, and 3207). For small data sets, the Z-score technique provides a method for determining genes that have significantly different expression in a single sample relative to other samples. These scores indicate the degree of deviation from the mean, in terms of the number of standard deviations, and they are likely to reveal well-behaving probes because they impose two requirements: a large deviation in one sample compared to the remaining samples, indicating a likely true biological difference in expression, and a small standard error, indicating that the probe set gives consistent measurements in the remaining n-1 samples. Before the Z-score analyses were applied, we computed geometric means of relevant replicate measurements for the two groups: FF tumor (4 patients, excluding sample 390) and FFPE-Agencourt tumor (4 patients, excluding sample 390). FFPE-Ambion tumor samples were not included in the Z-score analysis. We then computed Z-scores for each FFPE sample relative to the remaining three FFPE samples with a constant factor of 1 added to the denominator to avoid situations where the standard error was spuriously close to zero. For comparison, we also computed Z-scores using the same methodology for each of the FF samples relative to the other three FF samples. We then evaluated three criteria for filtering data from the FFPE samples. First, we considered all probes assayed on the array. Second, we considered only those probes where the Cy5 channel had a value of 1000 or greater ("1000 Cy5"). Finally, we considered top 100 differentially expressed probes that were both "1000 Cy5" and had the largest Z-scores, namely, the top 50 positive and top 50 negative Z-scores ("100 DE"). For each matched pair, we compared the selected FFPE Z-scores with corresponding FF Z-scores. Furthermore, for each of these three criteria, we evaluated correlation both quantitatively and qualitatively. The quantitative comparison measured the Pearson correlations of the Z-scores between the FFPE and FF samples. For the qualitative comparison, we tallied the "100 DE" probes whose signs of the FFPE Z-scores were the same as or opposite of the corresponding FF scores. The reported misclassification rates reflect the number of opposite-sign probes as a fraction of the total 100 probes.

Identification of genes that were differentially expressed in serous adenocarcinoma compared to normal ovary was achieved by a stepwise analysis. First, probes "passing" in all replicates of the four groups including FF, FFPE-Agencourt, and FFPE-Ambion tumors (see replicate details above) and FF normal samples (5 patients, 2 replicates per patient) were selected. Replicate Cy5/Cy3 ratios for each passing probe were averaged within a method before performing the Cyber t-test [26]. This t-test compared Cy5/Cy3 log_10 _ratios of FF tumor (n = 5) vs normal (n = 5) and FFPE tumor (n = 4) vs normal (n = 5); only probes with P values of equal to or less than 0.05 were considered further. All patient samples within a method were then averaged by calculating geometric means of the Cy5/Cy3 ratios for the genes that passed the two previous criteria ("passing" and t-test P value cutoff of 0.05). The resulting tumor-to-normal ratios are reported, where the ratio is at least 2-fold higher in all tumors than in all normals.

## Results

### Quality assessment of FF and FFPE samples

**Figure 1:**
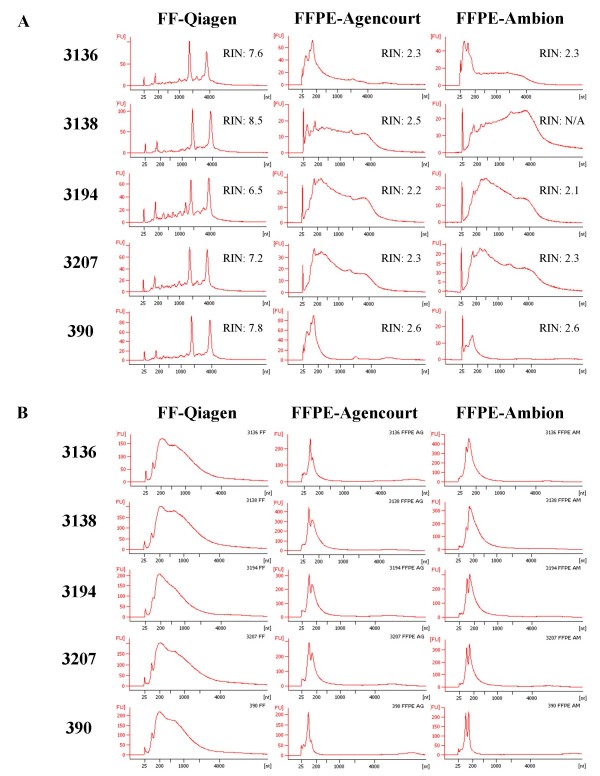
**Bioanalyzer profiles of total RNA (A) and cRNA (B) of matched FF and FFPE ovarian serous adenocarcinoma samples 3136, 3138, 3194, 3207 and 390**. The method used for RNA extraction (Qiagen, Agencourt, Ambion) is indicated for each sample type. The RNA Integrity Number (RIN) is shown next to each total RNA profile.

Following RNA quantification using Nanodrop, Agilent 2100 Bioanalyzer was utilized to generate RNA profiles for all FF and FFPE matched pairs (Figure 1A). FF samples showed high quality of RNA; 18S and 28S ribosomal peaks were present in all samples giving RNA Integrity Numbers (RINs) from 6.5 to 8.5. In contrast, the landmark ribosomal peaks were not detected in any of the FFPE samples, resulting in lower RINs. Since RINs depend on the presence of ribosomal peaks in the RNA samples, they can not accurately reflect the quality of FFPE RNA. For example, the most degraded sample 390 showed the highest RINs among all FFPE samples. RNA profiles obtained from the FFPE samples were similar for the two RNA extraction methods. FFPE samples 3138, 3194, and 3207 exhibited desirable profiles with elevated levels of high molecular weight RNAs. In contrast, small molecular weight RNAs were recognized as a sharp peak between 25 and 200 nucleotides (nt); the peak was the most prominent feature in samples 3136 and 390. Closer comparison between these two samples revealed that sample 3136 contained relatively low level of high molecular weight RNA fragments, while a flat electropherogram beyond 500 nt suggests absence of such RNA fragments in sample 390. Based on these results, FFPE sample 390 was classified as having inadequate RNA quality; this was surprising since slide examination indicated that it contained well preserved tissue.

**Figure 2:**
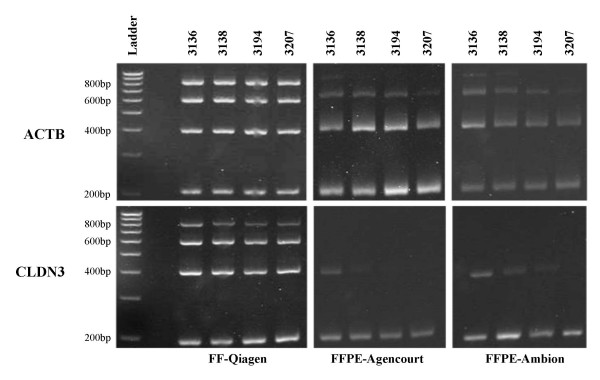
**RT-PCR amplification of different fragment sizes (200, 400, 600 and 800 bp) of *ACTB *and *CLDN3 *genes in FF and matched FFPE ovarian adenocarcinoma samples 3136, 3138, 3194, and 3207**. The method used for RNA extraction is indicated next to each sample type.

Additional quality assessment of total RNA obtained from matched FF and FFPE samples was done by RT-PCR amplification of different length fragments of *ACTB *and *CLDN3 *genes. As expected, RNA from FF samples resulted in amplification of all fragment sizes (Figure 2). The largest amplicon, 800 bp, was observed only in FF samples, suggesting that intact RNA of such length was rare in FFPE samples. RNA extracted from FFPE sample 390 failed to produce any *ACTB *and *CLDN3 *fragments (data not shown). For the remaining four FFPE samples, amplification of 200 bp, 400 bp and 600 bp fragments was achieved for the *ACTB *gene. For *CLDN3*, the 200 bp amplicon was detected in four FFPE samples. The 400 bp fragment was detected in two samples (3136 and 3138); one additional sample that was processed with the Ambion method (3194) showed weak presence of this amplicon, suggesting that it may have more intact RNA compared to the Agencourt counterpart. In contrast to *ACTB*, *CLDN3 *amplicons >400 bp were not detected in any of the FFPE samples.

cRNA yields from FF samples averaged at 12.5 μg, while those from FFPE samples ranged from 2.2 to 6.1 μg. This illustrates greater dependence of cRNA yield on RNA quality than on quantity since equal amounts of total RNA were used to generate cRNAs. High specific activities ranging from 10.9 to 13.6 pmol Cy5/μg were observed for cRNAs from FF samples. The corresponding FFPE samples resulted in noticeably lower specific activities ranging from 1.6 to 7.6 pmol Cy5/μg cRNA. FFPE sample 390 had the lowest dye incorporation compared to all other samples: 1.6 and 2.4 pmol Cy5/μg cRNA for Agencourt and Ambion, respectively. Based on this result, as well as on poor RNA quality described above, FFPE sample 390 was expected to perform poorly in subsequent expression profiling.

Similar to the quality assessment of total RNA, labeled cRNA was also examined on the 2100 Bioanalyzer (Figure 1B). The size distribution of cRNA was very different when comparing electropherograms obtained from FF and FFPE samples. In FF samples, cRNAs showed a wide profile encompassing molecular weight size above 4000 nt, with the highest frequency of fragments in ~200 nt range. In contrast, cRNAs generated from FFPE samples had narrow profiles of up to ~1000 nt, with the highest frequency of fragments in ~100 nt range. Similar to the initial total RNA profile, cRNA for sample 390 showed a very narrow distribution range and lacked fragments above ~500 nt.

### Gene expression profiling

**Figure 3:**
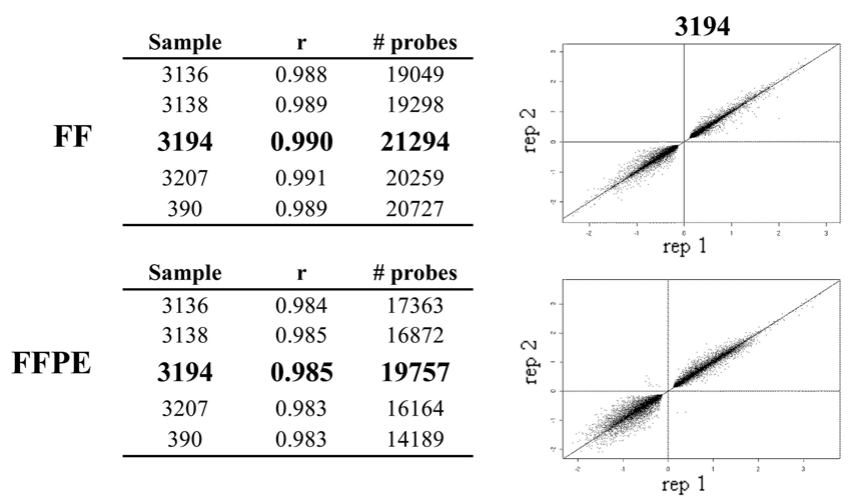
**Reproducibility of gene expression profiling within FF and FFPE sample type**. Pearson correlation value (r) and number of "passing" probes are listed for samples 3136, 3138, 3194, 3207 and 390. Scatter graphs of log_2 _ratios comparing two FF (top) and two FFPE replicates (bottom) are shown for sample 3194.

Reproducibility of genome-wide expression profiling of FF and FFPE samples was calculated using Pearson correlations (r). As shown in Figure 3, high reproducibility across replicates was observed regardless of the sample type. The r value for "passing" probes (see Methods) ranged from 0.988 to 0.991 within FF replicates and from 0.983 to 0.985 within FFPE replicates. Although both FF and FFPE samples displayed similar r values, scatter graphs obtained by plotting log_2 _ratio values of FF replicates appeared tighter compared to the FFPE replicates. The lowest number of passing probes was observed in FFPE sample 390; this was not surprising since this sample was classified as inadequate for expression profiling based on the quality of RNA and cRNA. Thus, sample 390 was not taken into account in subsequent analyses.

**Figure 4:**
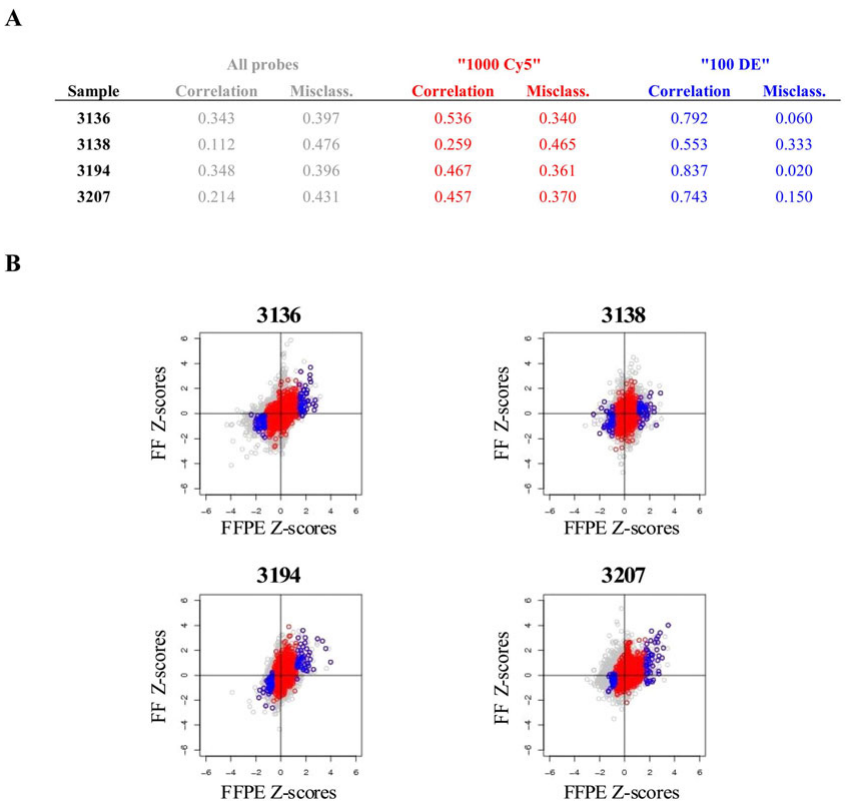
**Z-score analyses comparing FFPE and FF samples**. Pearson correlation and misclassification rate (**A**) as well as scatter plots of Z-score values (**B**) for samples 3136, 3138, 3194 and 3207 are shown for "All probes" (in grey), "1000 Cy5" (in red) and "100 DE" (in blue) probe selection criteria.

Z-score analysis was used to determine the level of concordance between matched FF and FFPE-Agencourt samples. As shown in Figure 4A, the three selection criteria showed progressively increasing correlations and decreasing misclassification rates, with the "100 DE" criterion achieving the best concordance. Sample 3138 showed the lowest concordance between its FF and FFPE z-scores, achieving a correlation of 0.553 and misclassification rate of 33%. However, the remaining samples showed relatively high correlations ranging from 0.743 to 0.837 and misclassification rates of 2–15%. The scatter plots of FF and FFPE Z-scores (Figure 4B) indicate that the three filtering criteria place tighter bounds on the analyses and the Z-scores for probe sets obtained under the "1000 Cy5" and "100 DE" criteria fall close to the diagonal axes. These two criteria also produce qualitatively better results with the differentially expressed probes found predominantly in the first and third quadrants of the plots.

Microarray gene expression profiles from ovarian tumor samples were also compared to those of normal ovary tissue. The analysis of four patient samples, including FF as well as FFPE samples processed by the Ambion and Agencourt methods, identified 56 genes whose expression was at least two-fold higher in tumor than in normal ovary. Table 1 lists tumor-to-normal ratios obtained from the FF and FFPE-Agencourt samples. Thirty three out of these 56 genes have been previously associated with various cancers and include cell cycle regulatory genes *CDC6 *[27], *CDT1 *[28] and *DTL *[29]. A well known marker of ovarian serous carcinoma, *CLDN3*, appeared at the top of the list. In contrast, the probe for CA125 was not classified as "passing" in all of the samples and thus it is not included in the list. Several other genes, including *TACSTD1 *[3], *PRAME *[30] and *ERBB4 *[31], have been linked to ovarian tumorigenesis. *PAX8 *has been recently reported as a useful marker for the differentiation between ovarian and metastasized breast carcinoma [32]. Finally, the remaining genes that we identified as overexpressed in tumor samples may potentially represent some novel markers of ovarian cancer.

**Table 1:** List of genes whose ratios are at least two-fold higher in ovarian serous adenocarcinoma compared to normal ovary.

**ProbeName**	**GeneName**	**Annotation**	**FF tumor/FF normal ratio**	**FFPE tumor/FF normal ratio**
**A_23_P91081**	***TACSTD1***	**tumor-associated calcium signal transducer 1 (TACSTD1), mRNA [NM_002354]**	**43.7**	**5.1**
A_23_P118894	*ATAD4*	ATPase family, AAA domain containing 4 (ATAD4), mRNA [NM_024320]	25.1	20.4
**A_23_P71017**	***CLDN3***	**claudin 3 (CLDN3), mRNA [NM_001306]**	**20.4**	**77.2**
A_23_P124335	*THC2539702*	Q9F8M7_CARHY (Q9F8M7) DTDP-glucose 4,6-dehydratase (Fragment), [THC2546981]	20.0	22.2
**A_24_P411186**	***BCL11A***	**B-cell CLL/lymphoma 11A (zinc finger protein) (BCL11A), transcript variant 1, mRNA [NM_022893]**	**18.7**	**14.8**
A_24_P273647	*LOC146439*	mRNA; cDNA DKFZp666L166 (from clone DKFZp666L166). [AL833749]	17.5	95.5
A_23_P393051	*C1orf172*	chromosome 1 open reading frame 172 (C1orf172), mRNA [NM_152365]	16.9	18.0
**A_24_P153713**	***MARVELD3***	**MARVEL domain containing 3 (MARVELD3), transcript variant 1, mRNA [NM_001017967]**	**14.4**	**34.7**
**A_24_P57977**	***SNIP***	**SNAP25-interacting protein (SNIP), mRNA [NM_025248]**	**12.6**	**32.1**
A_23_P83339	*RNF183*	ring finger protein 183 (RNF183), mRNA [NM_145051]	11.1	36.2
**A_23_P50096**	***TYMS***	**thymidylate synthetase (TYMS), mRNA [NM_001071]**	**11.1**	**3.0**
A_23_P33511	*AX721087*	Incyte sequence 47 from Patent WO0220754. [AX721087]	11.0	23.4
**A_23_P163481**	***BUB1B***	**BUB1 budding uninhibited by benzimidazoles 1 homolog beta (yeast) (BUB1B), mRNA [NM_001211]**	**10.2**	**4.3**
A_32_P57013	*BU540282*	AGENCOURT_10326456 NIH_MGC_141 cDNA clone IMAGE:6571686 5', mRNA [BU540282]	10.0	105.4
**A_23_P319859**	***EYA2***	**eyes absent homolog 2 (Drosophila) (EYA2), transcript variant 2, mRNA [NM_172113]**	**9.9**	**4.2**
**A_32_P183765**	***ERBB4***	**v-erb-a erythroblastic leukemia viral oncogene homolog 4 (avian) (ERBB4), mRNA [NM_005235]**	**9.6**	**8.6**
**A_23_P385861**	***CDCA2***	**cell division cycle associated 2 (CDCA2), mRNA [NM_152562]**	**9.4**	**6.8**
A_32_P46456	*THC2648851*	glucosidase, beta (bile acid) 2 (GBA2), mRNA [NM_020944]	9.4	22.0
A_32_P30874	*AJ420543*	mRNA full length insert cDNA clone EUROIMAGE 1090207. [AJ420543]	9.3	30.6
**A_24_P402588**	***BCL11A***	**B-cell CLL/lymphoma 11A (zinc finger protein) (BCL11A), transcript variant 1, mRNA [NM_022893]**	**9.2**	**31.1**
**A_23_P210001**	***PAX8***	**paired box gene 8 (PAX8), transcript variant PAX8A, mRNA [NM_003466]**	**8.4**	**31.3**
A_32_P136033	*AK090477*	mRNA for FLJ00399 protein. [AK090477]	8.4	12.8
A_23_P166508	*BC038245*	clone IMAGE:5241654, mRNA. [BC038245]	7.9	9.1
**A_23_P18579**	***PTTG2***	**pituitary tumor-transforming 2 (PTTG2), mRNA [NM_006607]**	**7.5**	**7.8**
A_23_P78958	*CAPS*	calcyphosine (CAPS), transcript variant 1, mRNA [NM_004058]	7.2	11.1
A_23_P72411	*CYP4X1*	cytochrome P450, family 4, subfamily X, polypeptide 1 (CYP4X1), mRNA [NM_178033]	7.2	4.0
A_32_P151244	*AK022268*	cDNA FLJ12206 fis, clone MAMMA1000941. [AK022268]	7.0	22.2
**A_23_P10385**^1^	***DTL***	**denticleless homolog (Drosophila) (DTL), mRNA [NM_016448]**	**6.5**	**5.4**
A_23_P68669	*CHODL*	chondrolectin (CHODL), mRNA [NM_024944]	6.4	9.6
A_32_P148745	*FLJ14712*	cDNA FLJ14712 fis, clone NT2RP3000825. [AK027618]	6.1	3.3
A_23_P151915	*GCNT3*	glucosaminyl (N-acetyl) transferase 3, mucin type. Acc:NP_004742] [ENST00000267857]	5.9	11.9
**A_32_P77933**	***FLJ22795***	**colon cancer-associated antigen AgSK1-2HT-ECS mRNA, complete cds. [AF316855]**	**5.9**	**24.1**
**A_23_P31073**	***MYB***	**v-myb myeloblastosis viral oncogene homolog (avian) (MYB), mRNA [NM_005375]**	**5.5**	**24.8**
**A_23_P28953**	***DNMT3B***	**DNA (cytosine-5-)-methyltransferase 3 beta (DNMT3B), transcript variant 6, mRNA [NM_175850]**	**5.3**	**8.9**
A_32_P117186	*CR749547*	mRNA; cDNA DKFZp686J17110 (from clone DKFZp686J17110) [CR749547]	5.0	4.1
**A_32_P128558**	***FLJ22795***	**colon cancer-associated antigen AgSK1-2HT-ECS mRNA, complete cds. [AF316855]**	**4.8**	**15.7**
**A_23_P166360**	***PRAME***	**preferentially expressed antigen in melanoma (PRAME), transcript variant 5, mRNA [NM_206956]**	**4.5**	**3.1**
**A_23_P103720**	***AGMAT***	**agmatine ureohydrolase (agmatinase) (AGMAT), mRNA [NM_024758]**	**4.3**	**2.8**
**A_23_P42935**	***BRAF***	**v-raf murine sarcoma viral oncogene homolog B1 (BRAF), mRNA [NM_004333]**	**4.2**	**3.6**
A_24_P741023	*BC008476*	cDNA clone IMAGE:4290767. [BC008476]	3.9	18.5
**A_23_P49972**	***CDC6***	**cell division cycle 6 homolog (S. cerevisiae) (CDC6), mRNA [NM_001254]**	**3.9**	**4.7**
A_24_P925191	*THC2640472*	Q2M1U4_HUMAN (Q2M1U4) Catalase, partial (31%) [THC2640472]	3.9	6.5
**A_24_P145316**	***DTNBP1***	**dystrobrevin binding protein 1 (DTNBP1), transcript variant 2, mRNA [NM_183040]**	**3.8**	**5.9**
**A_23_P19987**	***IGF2BP3***	**insulin-like growth factor 2 mRNA binding protein 3 (IGF2BP3), mRNA [NM_006547]**	**3.7**	**13.6**
A_23_P148768^1^	*F5*	coagulation factor V (proaccelerin, labile factor) (F5), mRNA [NM_000130]	3.6	78.1
**A_23_P37704**	***CDT1***	**chromatin licensing and DNA replication factor 1 (CDT1), mRNA [NM_030928]**	**3.5**	**7.5**
A_24_P544543	*CAPN1*	cDNA FLJ12257 fis, clone MAMMA1001501, highly similar to CALPAIN 1. [AK022319]	3.1	8.2
A_23_P89570	*ZMYND15*	zinc finger, MYND-type containing 15 (ZMYND15), mRNA [NM_032265]	2.9	11.8
**A_24_P340659**	***AF268613***	**POU 5 domain protein (POU5FLC1) mRNA, complete cds. [AF268613]**	**2.8**	**3.1**
**A_24_P343095**	***DHFR***	**dihydrofolate reductase (DHFR), mRNA [NM_000791]**	**2.8**	**2.6**
**A_24_P257099**	***DKFZp762E1312***	**hypothetical protein DKFZp762E1312 (DKFZp762E1312), mRNA [NM_018410]**	**2.7**	**2.9**
**A_23_P110957**	***FOXF2***	**forkhead box F2 (FOXF2), mRNA [NM_001452]**	**2.7**	**3.9**
**A_23_P75260**	***RASSF4***	**Ras association (RalGDS/AF-6) domain family 4 (RASSF4), mRNA [NM_032023]**	**2.6**	**7.4**
A_32_P81173	*USP34*	mRNA; cDNA DKFZp586J101 (from clone DKFZp586J101). [AL050376]	2.6	2.9
A_23_P156807	*SDHAP3*	succinate dehydrogenase complex, subunit A, flavoprotein pseudogene 3 (SDHAP3) [NR_003263]	2.6	9.5
**A_23_P109072**	***SALL4***	**sal-like 4 (Drosophila) (SALL4), mRNA [NM_020436]**	**2.6**	**3.5**
A_32_P132563	*POU5F1*	POU domain, class 5, transcription factor 1 (POU5F1), transcript variant 1, mRNA [NM_002701]	2.5	10.8
**A_23_P24870**^1^	***CD44***	**CD44 molecule (Indian blood group) (CD44), transcript variant 1, mRNA [NM_000610]**	**2.5**	**5.2**

Genes associated with cancer in previous reports are shown in bold. Fold change P≤0.05. Genes are sorted according to descending FF tumor/nomal ratios.

^1^Probe is represented on microarrays multiple times; average of multiple measurements is reported here.

## Discussion

FFPE samples are a desirable source of archival material for gene expression profiling studies due to their availability and the possibility of retrospective studies. At present, great variability is still being observed between gene expression profiles of matched FF and FFPE samples. Sample source and its classification, as well as the conditions used to fix and store samples, are some of many possible variables influencing gene expression. For example, a study relying on controlled fixation conditions to process bone marrow cells reported that FFPE samples appeared very similar to those of unfixed frozen equivalents [14]. However, controlled fixation procedure and use of cells may not represent an optimal approach to demonstrate the performance of archived FFPE tissue samples. Unsurprisingly, we have previously observed that FFPE cell pellets dissociated faster during Proteinase K digestion and often resulted in better RNA quality compared to FFPE tissues.

FFPE samples are routinely prepared by fixing tissues in 10% neutral-buffered formalin for 12 to 24 hours at ambient temperature. The FFPE samples used in this study, except for sample 390, were fixed in formalin for 4 to 18 hours at 4°C. These samples appear to perform better than sample 390 which was fixed using routine conditions. Thus, it appears that shorter time and lower temperature of fixation can significantly affect FFPE sample quality. Other variables such as size of collected tissue, time elapsed to fixation and storage time could have further affected this outcome [33,34]. Regarding storage time, we have noticed that ribosomal RNA peaks could be detected in FFPE samples that were stored properly for up to one year and not for longer periods of time (data not shown).

Two commercially available FFPE RNA isolation kits, Ambion Optimum and Agencourt FormaPure, were tested here for extracting RNA from five FFPE ovarian serous adenocarcinoma samples. Sufficient amounts of total RNA for gene expression profiling were achieved by processing two to eight FFPE sections per patient. Despite pooling multiple sections, the RNA yields from FFPE samples were always significantly lower compared to those obtained from FF samples (data not shown). In addition, the obtained RNA amount was not proportional to the surface area of tissues used for RNA extraction. Although significant necrosis was not detected in any of the sections, we noticed differences in tissue density and composition. For example, presence of vasculature and adipose was detected in some sections and undoubtedly affected the amounts of recovered nucleic acids. With respect to tissue content, all FFPE samples used in this study were required to contain 70–85% tumor cells, ensuring that they truly represented ovarian serous adenocarcinomas.

Two quality control assays, the Agilent 2100 Bioanalyzer and RT-PCR, were employed to assess the integrity of RNA obtained from FF and FFPE samples. Although RNA profiles from FFPE samples lacked well defined 18S and 28S ribosomal peaks, the method was successful in identifying inadequate samples, such as sample 390, containing predominantly small molecular weight fragments (<200 nt). Our RT-PCR assay tested the RNA for the presence of different size fragments of two genes, *ACTB *and *CLDN3*. Not surprisingly, we were not able to amplify any fragments in FFPE sample 390. Thus, the data synergy observed between the Bioanalyzer and the RT-PCR assay proved to be very useful in qualifying FFPE samples suitable for gene expression profiling on microarrays. These two assays also demonstrated that both the Ambion Optimum and the Agencourt FormaPure methods were successful in obtaining RNA of similar quality. Together, the combination of quality control methods used here should be effective in recognizing poor-performing FFPE samples and could be used to prevent unnecessary array hybridizations. A different method for identifying unacceptable FFPE samples has been described by NuGEN Technologies [35].

Gene expression profiling data of ovarian tumors demonstrated high correlations between replicate FF (≥0.988) and FFPE samples (≥0.983), illustrating good reproducibility of each method. Although reproducible, gene expression profiling of FFPE samples is affected by reduced number of "passing" probes (33–46% compared to 44–49% in FF) and thus detects fewer differentially expressed genes compared to FF samples. Lassmann *et al*. recently reported similar findings by detecting 36% and 50% of probes for FFPE and FF samples, respectively [20]. Our comparison between FF and their matched FFPE samples revealed the highest concordance of 0.84 for sample 3194. Although not directly comparable, a mean concordance of 0.86 was reported previously when comparing matched FF and FFPE samples on a different microarray platform [17]. Haque *et al*. reported a correlation of 0.65 between unmatched FF and FFPE pediatric glioblastomas [23]. While the magnitude of differential expression in FFPE samples might not be accurate, our Z-score analysis indicated that the direction of the change was correct in most cases, as demonstrated by low misclassification rates.

The comparison of ovarian serous adenocarcinoma to normal ovary identified 56 genes that are overexpressed in both FF and FFPE tumor samples. Several genes among them, including *CLDN3*, were previously recognized for their roles in ovarian tumorigenesis, [2-5,30-32]; additional genes with unknown roles were also identified. Profiling of archival FFPE samples has been used previously to identify gene signatures that may serve as prognostic and diagnostic markers [20-24]. Regarding ovarian cancer, a set of 86 gene signatures that seems to predict overall survival was recently identified by microarray profiling [36]. Furthermore, 57 of these 86 genes were confirmed in an independent dataset [37]. Together, these findings suggest that archival FFPE samples can be successfully used to identify potentially novel disease markers. At the same time, it is recognized that gene expression profiling of FFPE samples on microarrays has some limitations. In our study, as well as in Van Deerlin *et al*. [38], the magnitude of differential expression was typically higher in FFPE than in FF samples, suggesting higher level of noise in the FFPE data. Therefore, the elimination of false positives and identification of subtle changes in gene expression in FFPE samples remain challenging, especially in studies lacking FF counterparts.

Technological improvements in handling FFPE samples are constantly evolving; some of them clearly lead towards better quality of microarray expression data. One such improvement entails a change in primers used during amplification. At present, commonly used procedures rely on oligo (dT) primers which introduce 3' end bias [17,39]; consequently, most commercial microarrays have probes designed within the last several hundred bases of each transcript. A new amplification procedure, developed recently by NuGEN, utilizes random primers in addition to oligo (dT) primers, thus alleviating the 3' end bias. Initial studies suggest that such whole transcript amplification provides a significant advantage when processing FFPE samples [14,20]. Thus, this amplification method deserves further investigation and holds promise for improving the performance of FFPE samples in future microarray profiling studies.

## Conclusion

Five matched FFPE and FF ovarian tumor samples were profiled on microarrays, illustrating the level of gene expression similarity between the two sample types. Ovarian tumor and normal samples were also compared, identifying a set of differentially expressed genes characteristic of ovarian adenocarcinoma. Conclusively, our study demonstrates that archived clinical samples, such as FFPE ovarian adenocarcinomas, represent a valuable source for genome-wide expression profiling and can be successfully used for the identification of potentially novel carcinoma markers. Further improvements in FFPE sample handling and new amplification approaches hold promise for even better performance of FFPE samples in future microarray studies.

## Competing interests

The authors declare that they have no competing interests.

## Authors' contributions

GF designed and carried out microarray experiments, SG and TDW performed data analyses and ZM conceived and directed the project. GF and ZM wrote the manuscript.

## Pre-publication history

The pre-publication history for this paper can be accessed here:


